# Acquired defects in CFTR-dependent β-adrenergic sweat secretion in chronic obstructive pulmonary disease

**DOI:** 10.1186/1465-9921-15-25

**Published:** 2014-02-25

**Authors:** Clifford A Courville, Sherry Tidwell, Bo Liu, Frank J Accurso, Mark T Dransfield, Steven M Rowe

**Affiliations:** 1 Department of Medicine, MCLM 706, 1918 University Blvd, University of Alabama at Birmingham, Birmingham, AL, USA; 2 Department of Pediatrics, University of Alabama at Birmingham, Birmingham, AL, USA; 3 Department of Pediatrics, University of Colorado, Aurora, CO, USA; 4 Department of Cell Developmental and Integrative Biology, University of Alabama at Birmingham, Birmingham, AL, USA; 5 The Gregory Fleming James Cystic Fibrosis Research Center, University of Alabama at Birmingham, Birmingham, AL, USA; 6 UAB Lung Health Center, University of Alabama at Birmingham, Birmingham, AL, USA

## Abstract

**Rationale:**

Smoking-induced chronic obstructive pulmonary disease (COPD) is associated with acquired systemic cystic fibrosis transmembrane conductance regulator (CFTR) dysfunction. Recently, sweat evaporimetry has been shown to efficiently measure β-adrenergic sweat rate and specifically quantify CFTR function in the secretory coil of the sweat gland.

**Objectives:**

To evaluate the presence and severity of systemic CFTR dysfunction in smoking-related lung disease using sweat evaporimetry to determine CFTR-dependent sweat rate.

**Methods:**

We recruited a cohort of patients consisting of healthy never smokers (N = 18), healthy smokers (12), COPD smokers (25), and COPD former smokers (12) and measured β-adrenergic sweat secretion rate with evaporative water loss, sweat chloride, and clinical data (spirometry and symptom questionnaires).

**Measurements and main results:**

β-adrenergic sweat rate was reduced in COPD smokers (41.9 ± 3.4, P < 0.05, ± SEM) and COPD former smokers (39.0 ± 5.4, P < 0.05) compared to healthy controls (53.6 ± 3.4). Similarly, sweat chloride was significantly greater in COPD smokers (32.8 ± 3.3, P < 0.01) and COPD former smokers (37.8 ± 6.0, P < 0.01) vs. healthy controls (19.1 ± 2.5). Univariate analysis revealed a significant association between β-adrenergic sweat rate and female gender (β = 0.26), age (−0.28), FEV_1_% (0.35), dyspnea (−0.3), and history of smoking (−0.27; each P < 0.05). Stepwise multivariate regression included gender (0.39) and COPD (−0.43) in the final model (R^2^ = 0.266, P < 0.0001).

**Conclusions:**

β-adrenergic sweat rate was significantly reduced in COPD patients, regardless of smoking status, reflecting acquired CFTR dysfunction and abnormal gland secretion in the skin that can persist despite smoking cessation. β-adrenergic sweat rate and sweat chloride are associated with COPD severity and clinical symptoms, supporting the hypothesis that CFTR decrements have a causative role in COPD pathogenesis.

## Introduction

The defining feature of chronic obstructive pulmonary disease (COPD) is airflow limitation, although it is recognized that the disease exhibits heterogeneous pathologic features in the lung, including mucus retention [1,2]. Acquired cystic fibrosis transmembrane conductance regulator (CFTR) dysfunction caused by cigarette smoke exposure has been implicated as a potential factor in the pathogenesis of COPD by causing abnormalities in the mucociliary transport apparatus [3-8]. Cigarette smoke inhibits CFTR activity in airway cells *in vitro*[3,5,6,9,10], and in human subjects with and without COPD [3,9], indicating a potential therapeutic target to combat mucus stasis in the disease. Further studies have shown that smoking-related CFTR dysfunction and its downstream effects, such as depleted airway surface liquid depth or delayed mucociliary transport, can be reversed in vitro by augmentation of CFTR dependent anion transport by the CFTR potentiator ivacaftor [9] or by osmotic loading with hypertonic saline [10], providing further evidence of the biologic and therapeutic relevance of this pathway.

While several studies have established the presence of acquired CFTR dysfunction in the COPD airway, it has only been recently reported that the defect in CFTR function may extend beyond the airways. Raju *et al*. recently described a cohort of current and former smokers with and without COPD who each exhibited elevated sweat chloride when compared with healthy never smokers, suggesting a decrement in CFTR function outside the respiratory tract [11]. In addition, this study demonstrated that inhaled whole cigarette smoke decreased CFTR activity in the distal ileum of mice, confirming similar findings from rectal biopsy samples of human smokers [11]. These findings support the notion that CFTR decrements may be a systemic phenomenon in smoking related diseases.

An effort to detect the degree of systemic impairment of CFTR in acquired disorders may be limited by traditional assays such as quantitative pilocarpine iontophoresis (QPIT) which may not be maximally sensitive towards detecting modest decrement in CFTR function [12]. Further, QPIT measures the impact on the absorptive activity of CFTR, rather than the secretory activity, which may be a substantial contributor to pulmonary pathology [13]. Recently Quinton and colleagues developed an assay that may be more sensitive for detecting a modest decrement in CFTR function by using quantitative evaporimetry to measure ß-adrenergic-induced sweat secretion rate [14]. The assay provides a direct assessment of CFTR activity in the sweat gland, which may correlate better with secretory gland function, including airway submucosal glands, which are markedly abnormal in CF and could be a significant contributor to pathogenesis in COPD [15,16]. To confirm our hypothesis that acquired CFTR dysfunction can be detected beyond the airway and test whether the defect alters function of the glandular compartment, we used this assay in patients without CFTR mutations to quantify glandular CFTR activity while also assessing the phenotypic expression of COPD. Our results indicate a systemic reduction in CFTR function in COPD patients as detected by both evaporimetry and sweat chloride, which has significant physiologic and clinical implications.

## Methods and materials

### Recruitment of human subjects

Protocols were approved by the University of Alabama at Birmingham’s Institutional Review Board and all subjects provided written informed consent (Approval F101110001). Subjects age 40–75 years were eligible. Subjects were categorized into one of four eligible groups: (1) healthy never smokers; (2) healthy smokers with ≥ 10 pack-year smoking history who continued to smoke more than 10 cigarettes per day; (3) COPD patients with ≥ 10 pack-years who continued to smoke more than 10 cigarettes daily; (4) COPD patients with ≥ 10 pack years who have remained abstinent from tobacco for more than 1 year. Subjects were recruited prospectively and disease status was not assigned until after enrollment. More detailed inclusion and exclusion criteria are detailed online (Additional file 1).

### Phenotype characterization

The presence of chronic bronchitis (as assessed by the Medical Research Council definition of cough and sputum present most days of three months in two consecutive years) [17] and COPD symptom severity (measured by the Breathlessness Cough and Sputum Scale questionnaire) were documented on each subject [18]. COPD severity was also measured by components of the BODE index (body mass index, obstruction (FEV_1_% predicted), and dyspnea (Modified Medical Research Council (MMRC) Questionnaire)) [19]. Cumulative cigarette exposure was recorded as total lifetime pack-years smoked and current cigarette use. Smoking status of never smokers and COPD former smokers was confirmed with urine cotinine analysis.

### Sweat secretion test

We performed sweat evaporimetry to measure exocrine gland function of the sweat gland on all subjects [14]. All evaporimetry tracings were analyzed by a single blinded investigator. Sweat rates were calculated as the maximal stable rate observed after β-adrenergic injection less the lowest observed rate after the preceding atropine injection, as previously described [14]. The rate of evaporative water loss (kg water loss/m^2^/h) is expressed as evaporimeter units.

### Genetic testing

Genetic testing (50 mutation analysis) for CFTR mutations was performed on all participants by using a commercially accredited facility (Baylor Medical Genetics, Houston, TX). Patients with a CFTR mutation were excluded from the analysis.

### Sweat chloride testing

Sweat chloride was measured by quantitative pilocarpine iontophoresis via the Macroduct (Westcor Inc., Logan, UT) collection system as previously reported [11,20,21].

### Statistics

Sweat rate, sweat chloride, and subgroup analyses were compared using ANOVA. Post-hoc tests for multiple comparisons were calculated using Fisher’s least significant difference if ANOVA was significant. All statistical tests were two-sided and performed at a 5% significance level using GraphPad Prism (La Jolla, CA). Error bars designate SEM unless indicated otherwise. Regression analyses were performed using SPSS (IBM, Armonk, NY); multiple regression was conducted using a step-wise approach.

## Results

**Figure 1:**
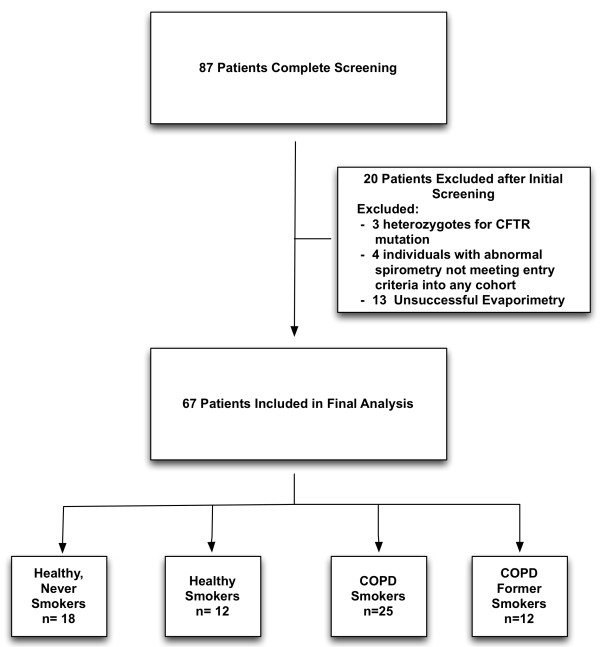
Subject disposition.

We recruited 87 subjects and 20 were excluded based on presence of CFTR genetic mutation, unsuccessful sweat rate assay, or inadequate disease categorization based on history or spirometry (Figure 1). The summary of the patients included in the final analysis is presented in Table 1. There was a slight predominance of males in each group but the balance was not significantly different in any cohort. The mean age of the COPD patients was significantly greater than the other groups. As expected, the BCSS and MMRC scores were significantly greater and FEV_1_ significantly lower in the COPD cohorts.

**Table 1:** Summary data describing study subjects undergoing sweat evaporimetry

		**Healthy never smokers**	**Healthy active smokers**	**COPD active smokers**	**COPD former smokers**	**P Value**	**Pairwise with P < 0.05**
						**(ANOVA)**	
**N**		18	12	25	12		
**Age (mean, SD)**		47 ± 9	52 ± 8	58 ± 9	68 ± 8	< 0.001	HNS-CS, HNS-CFS, HS-CFS, CS-CFS
**Gender N (%)**							
	**Male**	11 (61%)	6 (50%)	19 (76%)	9 (75%)	0.387	
	**Female**	7 (39%)	6 (50%)	6 (24%)	3 (25%)		
**Race N (%)**							
	**Caucasian**	13 (72%)	4 (27%)	14 (56%)	8 (67%)	0.185	
	**African-American**	5 (28%)	8 (63%)	11 (44%)	4 (33%)		
**Chronic bronchitis N (%)**		0 (0%)	2 (18%)	13 (52%)	6 (50%)	< 0.001	HNS-CS, HNS-CFS, HS-CS
**Spirometry**							
	**FEV1 (L) (Mean, SD)**	3.55 ± 1.02	2.92 ± 0.82	2.03 ± 0.73	1.23 ± 0.40	< 0.001	HNS-CS, HNS-CFS, HS-CS, HS-CFS, CS-CFS
	**FEV**_**1**_**% pred (Mean, SD)**	1.03 ± 0.10	0.97 ± 0.12	0.60 ± 0.16	0.41 ± 0.18	< 0.001	HNS-CS, HNS-CFS, HS-CS, HS-CFS, CS-CFS
**Pack-years (Mean, SD)**		0	31.08 ± 14	38 ± 19	44 ± 19	<0.0001	HNS-HS, HNS-CS, HNS-CFS
**MMRC (Mean, SD)**		0.06 ± .236	0.83 ± .577	1.96 ± 1.197	2.83 ± 1.03	< 0.001	HNS-HS, HNS-CS, HNS-CFS, HS-CS, HS-CFS, CS-CFS
**BCSS (Mean, SD)**		0.06 ± 0.236	0.75 ± 0.965	3.46 ± 3.007	2.67 ± 2.43	< 0.001	HNS-CS, HNS-CFS, HS-CS, HS-CFS

MMRC = Modified Medical Research Council criteria; BCSS = Breathlessness, Cough and Sputum Scale. The mean and standard deviation for clinical characteristics for our patient cohorts are provided. Post-hoc pairwise analysis was done using least significant difference as appropriate. HNS = Healthy Never Smokers; HS = Healthy Smokers; CS = COPD Active Smokers; CFS = COPD Former Smokers.

**Figure 2:**
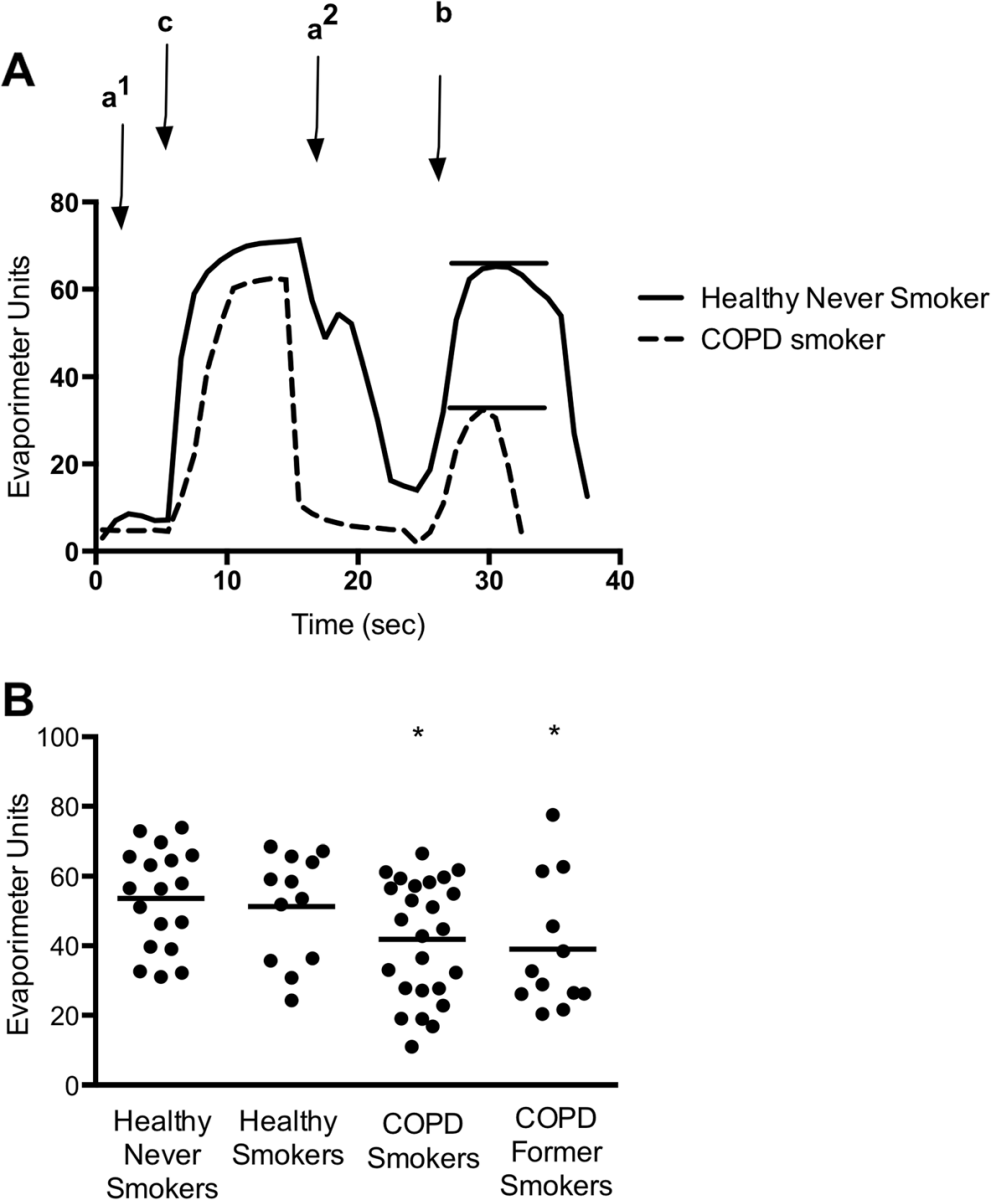
**Systemic CFTR dysfunction in COPD subjects as demonstrated by β-Adrenergic sweat secretion rate. (A)** Representative sweat secretion assay tracings of a healthy never smoker (solid) and a COPD patient (dotted). Evaporative water loss is measured continuously following injection of (a^1^) atropine, (c) carbachol, (a^2^) atropine again, and (b) β-adrenergic cocktail stimulus. The stable β-adrenergic stimulated sweat secretion rate is marked with a bar. **(B)** Summary data plotting maximal β-adrenergic sweat rate for each individual subject. *P < 0.05.

Evaporimetry was successfully performed on 67 eligible subjects. Representative evaporimeter tracings are shown in Figure 2A. In COPD subjects, there was a significant decrement in the β-adrenergic-induced sweat rate, but not the cholinergic sweat rate. The mean β-adrenergic sweat rate comparisons are presented in Figure 2B. The mean (± standard error) β-adrenergic sweat rate for healthy, never smokers was 53.6 ± 3.3. This rate was significantly lower in COPD smokers (41.9 ± 3.4; P < 0.05), and COPD former smokers (39.0 ± 5.4; P < 0.05). There was a trend toward reduced sweat rate in healthy smokers, 51.3 ± 4.4, when compared with healthy never smokers, but this difference was not statistically significant.

**Figure 3:**
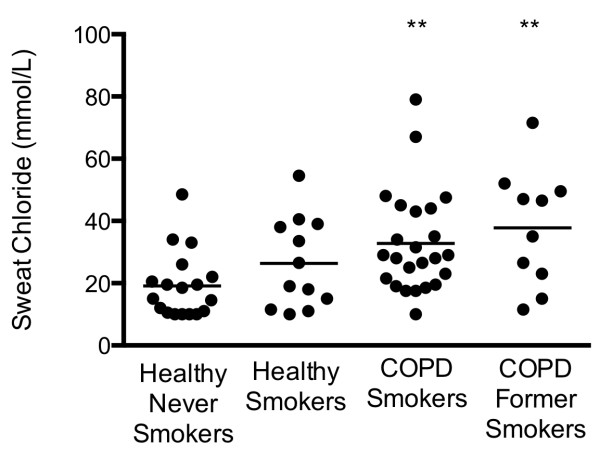
**Sweat chloride abnormality in COPD subjects.** Quantitative pilocarpine iontophoresis measured concurrently to the β-adrenergic sweat test in each subject. **P < 0.01.

Sweat chloride testing revealed a similar pattern of abnormalities as detected by β-adrenergic sweat rate testing (Figure 3). The mean sweat chloride for healthy never smokers was 19.1 ± 2.4 whereas the concentrations in COPD smokers (32.8 ± 3.3; P < 0.01) and COPD former smokers (37.8 ± 6.0; P < 0.01) were significantly increased and generally consistent with previous results [11]. There was a trend towards elevated sweat chloride in healthy smokers (mean of 26.38 ± 4.19) but this was not statistically different than the control group.

**Figure 4:**
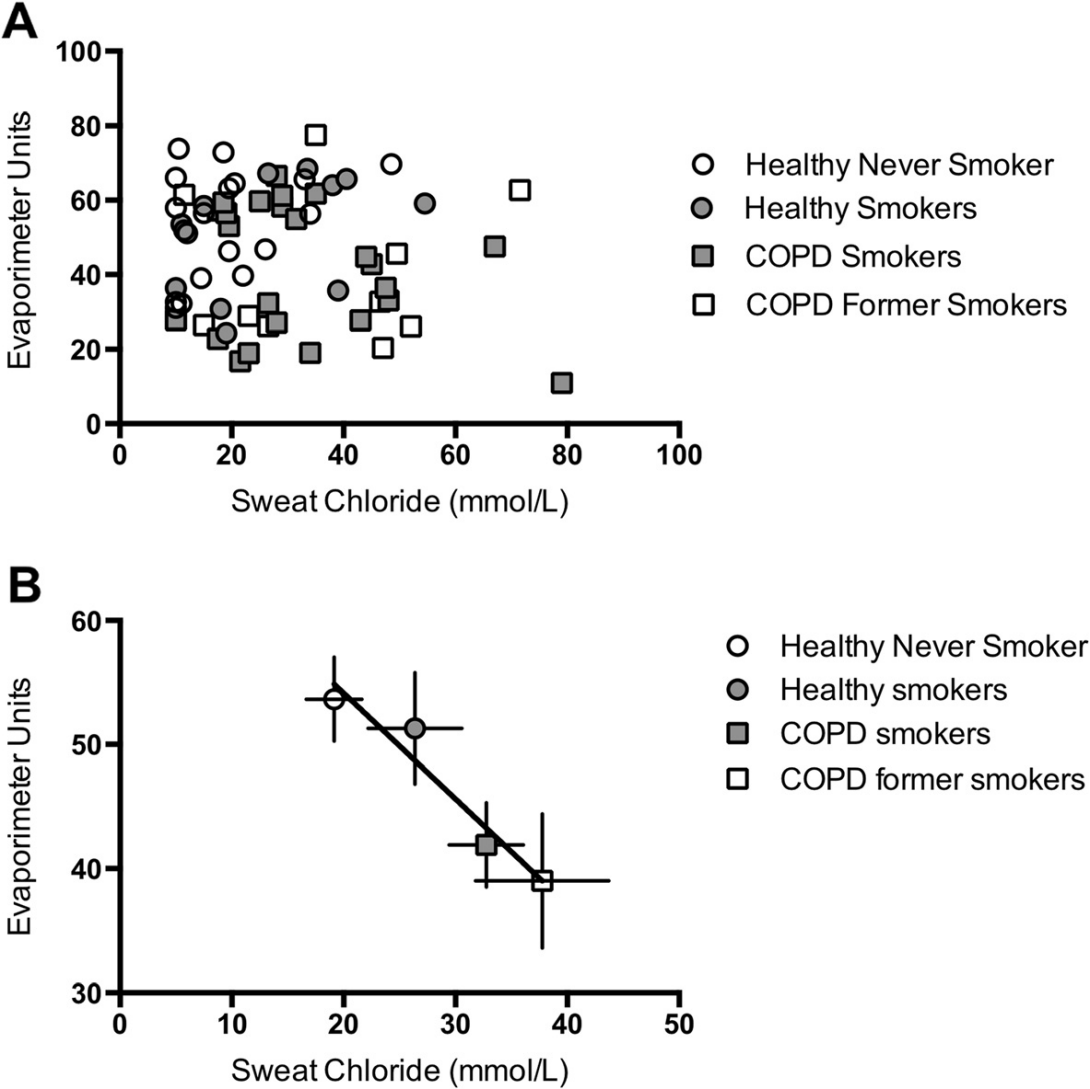
**Relationship between sweat chloride and β-adrenergic sweat rate.** Sweat chloride (x-axis) and β-adrenergic sweat rate (y-axis) are plotted for each individual subject **(A)** and by disease group **(B)**. Regression line shown in **(B)** was statistically significant (R^2^ = 0.933, P < 0.05).

Sweat chloride and ß-adrenergic sweat rate did not correlate on an individual basis when the entire study population was included (Figure 4A). When the two assays were compared after grouping patients based on disease group, a linear correlation was readily apparent (Figure 4B; P < 0.05, R^2^ = 0.93).

Regression analysis was performed to evaluate which clinical parameters were associated with reduced CFTR-dependent sweat secretion (Table 2). As described previously, females exhibited lower sweat rates than males [14]. Univariate analysis also indicated a negative association with age. Abnormal sweat rate was also associated with features of COPD severity, including dyspnea as assessed by MMRC and reduced FEV_1_%, as well as with any history of smoking. Stepwise multivariate regression was performed with all available clinical covariates. The final model included gender (β = 0.39) and COPD (β = −0.43), indicating a pronounced effect of COPD on β-adrenergic sweat rate, even when controlled for gender (R^2^ = 0.266, P < 0.0001). An adjusted analysis with gender included as a covariate demonstrated that COPD patients exhibited significantly lower CFTR-dependent sweat rate than non-COPD subjects (Table 3). An adjusted analysis that included age and gender did not significantly alter conclusions.

**Table 2:** Univariate regression analysis in relation to β-adrenergic sweat rate

	**Beta**	**95% Confidence intervals**	**P-value**
**Gender**	0.259	2.09, 19.23	**0.016**
**Age**	−0.282	−0.812, -.069	**0.021**
**Race**	0.062	−6.40, 10.64	0.621
**BCSS**	−0.135	−2.71, 0.801	0.282
**Bronchitis**	−0.148	−14.466, 3.635	0.236
**FEV1**	0.345	6.820, 35.08	**0.004**
**MMRC**	−0.3	−6.877, -0.789	**0.014**
**Pack years**	0.017	0.257, 0.289	0.906
**Active smoke**	−0.093	−11.570, 5.255	0.456
**Ever smoke**	−0.271	−19.225, -1.233	**0.026**
**BMI**	−0.02	−0.813, 0.691	0.872
**Cigarette/day**	−0.188	0.651, 0.084	0.128

MMRC = Modified Medical Research Council criteria; BCSS = Breathlessness, Cough and Sputum Scale; BMI = Body Mass Index.

**Table 3:** Gender-adjusted mean sweat rates

	**β-Adrenergic sweat rate**	**95% Confidence interval**
**Healthy never smokers**	54.47 ± 3.60	47.28, 61.65
**Healthy smokers**	53.64 ± 4.45	44.75, 62.53
**COPD smokers**	40.70 ± 3.07 **	34.57, 46.83
**COPD former smokers**	37.93 ± 4.41**	29.13, 46.73

Mean ± SEM is presented for each group following adjustment for gender. **P < 0.01.

We also analyzed the relationship between clinical parameters and sweat chloride. Univariate analysis revealed a significant relationship between sweat chloride abnormality and age, COPD, FEV_1_%, COPD symptoms, smoking status and intensity, and decreased BMI (Table 4). These findings were largely consistent with a previous study [11]. Multiple linear regression using the stepwise approach included only smoking intensity (pack-years) in the final model (R^2^ = 0.185, P < 0.001). The effect of smoking intensity persisted (β = 0.280, R^2^ = 0.224, P = 0.051), even when age was included as a covariate, excluding its contribution to the abnormality. For further confirmation that age was not contributing to our findings, we examined the relationship between age and sweat chloride in normal non-smoking controls using all individuals in our COPD study database (N = 33), including those presented in a previous publication [11]. We found no significant relationship between age and sweat chloride (β = 0.062, P = 0.731, N = 33). Given this effect, for every 10 years of age, sweat chloride would only be expected to increase by 0.6 mmol/L, an insufficient magnitude to explain our results based on age alone.

**Table 4:** Univariate regression analysis in relation to sweat chloride

	**Beta**	**P-value**
**Gender**	0.141	0.268
**Age**	0.416	**0.001**
**Race**	0.031	0.808
**BCSS**	0.359	**0.004**
**Bronchitis**	0.177	0.174
**FEV**_**1**_**%**	−0.309	**0.013**
**MMRC**	0.312	**0.013**
**Pack years**	0.429	**<0.001**
**Active smoker**	0.183	0.149
**Ever smoke**	0.396	**0.001**
**BMI**	−0.250	**0.047**
**Cigarette/day**	0.428	**<0.001**
**COPD**	0.380	**0.002**

MMRC = Modified Medical Research Council criteria; BCSS = Breathlessness, Cough and Sputum Scale; BMI = Body Mass Index.

## Discussion

We describe the first use of ß-adrenergic sweat secretion to detect CFTR dysfunction in individuals with smoking-related lung disease providing strong evidence that COPD is associated with acquired CFTR dysfunction in extra-pulmonary organs. β-adrenergic-induced sweat rate has recently been established as a potential diagnostic option in those who possess modest CFTR abnormalities [14]. The use of evaporimetry in this setting allows assessment of exocrine function and may carry greater clinical significance compared to traditional testing since it has been suggested that CFTR abnormalities in glandular epithelia and other secretory compartments are highly relevant to lung pathophysiology [14]. Acquired CFTR abnormalities in the sweat gland epithelium are consistent with altered sweat chloride identified in smokers and COPD patients derived from a larger cohort of individuals with the exact same clinical inclusion criteria [11].

Our observations have important implications on the relationship between CFTR dysfunction and COPD pathogenesis. The results demonstrate that CFTR dysfunction is present in a subset of COPD patients and can be readily detected beyond the airway using a simple and inexpensive clinical test in both current and former smokers with the disease. While studies have demonstrated that CFTR function can recover in the nasal epithelium [7,9] and that nasal mucociliary clearance can recover following smoking cessation in healthy smokers without COPD [22], our results suggest CFTR deficits can be sustained in individuals with a diagnosis of COPD. A similar finding was observed in our prior analysis which also included intestinal current measurements of former smokers [11]; a trend was also seen in the measurement of CFTR function in lower airways of COPD former smokers [8]. Moreover, Bodas *et al.* reported reduced CFTR expression was also observed in the lungs of patients with emphysema despite smoking cessation [23].

The current cohort indicates that the CFTR abnormality is also associated with disease phenotype, further supporting a causal association. Sloane *et al*. demonstrated reduced CFTR–dependent nasal potential difference (NPD) in COPD subjects, and that CFTR dysfunction was associated with symptoms of chronic bronchitis, even when controlled for smoking [9]. This association was noted in the lung by Dransfield *et al.*[8]. Smoking is associated with a defect in nasal mucociliary clearance, which correlates with smoking intensity and chronic bronchitis symptoms [22]. These reports are consistent with the present data that demonstrate an association between abnormal sweat gland function and COPD symptoms (dyspnea), and suggest sweat testing may be a feasible means to detect individuals with CFTR-dependent abnormalities in physiology due to COPD or cigarette smoking.

Persistence of CFTR dysfunction among COPD former smokers suggests the presence of a sustained mediator of CFTR dysfunction in COPD, a concept proposed by Raju *et al.*[11], although the exact mechanism by which reduced CFTR function is conferred in COPD is not yet fully elucidated. It has been demonstrated that cigarette smoke extract can have an impact on basolateral ion transport [5], which in turn could impact CFTR function at the airway surface by disrupting electrochemical gradients. More recent work by Moran *et al.* suggests that cigarette smoke can impact CFTR in a mechanism similar to CFTR inhibitors in the arylaminobenzoate class and may affect CFTR via access through the cytosol, a pathway consistent with entry via the systemic circulation [24]. These findings provide further understanding of how a tobacco constituent in the serum could affect apical CFTR function, either within the airway or sweat gland lumen.

While there may be many cigarette smoke constituents that contribute to acquired CFTR dysfunction [25], one such agent found to circulate in smokers was the cigarette smoke constituent and inflammatory byproduct acrolein, a reactive aldehyde that directly inhibits CFTR open channel probability in isolated membrane patches and blocks CFTR mediated short-circuit current in epithelial monolayers [11]. Interestingly, the effects of acrolein can be sustained due to retention in tissue [26], and is known to induce ER stress, which can alter CFTR expression [27]. While a number of studies have established that CFTR dysfunction can be induced in the airways in response to direct insults from cigarette smoking [3,9,10] (e.g. cadmium [25,28]), arsenic [29], hypoxia [4] or inflammatory byproducts (e.g. neutrophil elastase) [8,30], whether these alternative mediators are also operative in the systemic circulation has not yet been defined. Other studies have elucidated potential serum biomarkers for COPD including interleukin-6, C-reactive protein, tumor necrosis factor-α, among others [31-33], which support the notion that COPD causes a low-grade systemic inflammatory state. Similarly, systemic CFTR dysfunction could explain phenotypic abnormalities more common in individuals who smoke, such as reduced BMI [34], idiopathic pancreatitis [35], male infertility [36], and osteoporosis [37], since each of these are definitively linked to CFTR physiology and are expressed in individuals with varying degrees of genetic CFTR abnormality. Further studies to define the pathogenic role of acquired systemic CFTR dysfunction in this disease are needed to better understand the role of this pathway.

Our study serves to validate recent applications of sweat rate in humans with various severities of genetic CFTR mutations since patients with smoke exposure or COPD exhibit partial CFTR decrements akin to individuals who are heterozygous for CFTR mutations. As in prior studies, we used sweat chloride as a means to confirm the β-adrenergic sweat secretion assay. Though sweat rate and sweat chloride did not correlate on an individual basis (Figure 4A), a linear relationship was observed across disease groups (Figure 4B). While the lack of correlation among individuals could be due to the relatively small number of subjects in each group, recent studies in CF have indicated that altered sweat chloride and response to therapeutics are heterogeneous among individuals [21,38]; poor correlation between CFTR biomarkers (i.e. sweat chloride versus NPD) in the same individuals has also been problematic in these studies, which may explain why a tighter relationship is not apparent in this sample.

**Figure 5:**
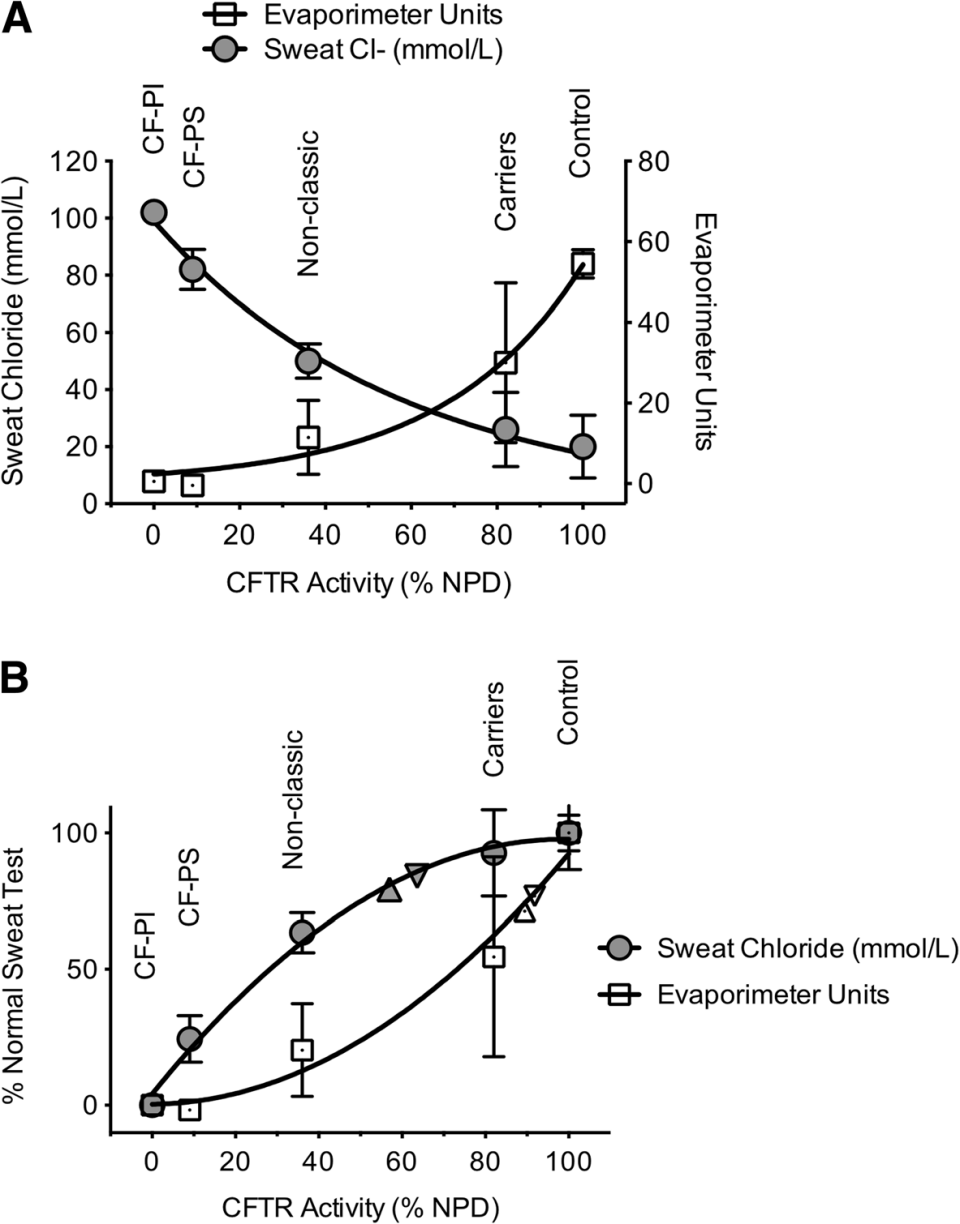
**Relationship between sweat tests and CFTR function estimated by nasal potential difference. (A)** Based on previously reported values from the literature, sweat chloride and β-adrenergic Sweat Rate are plotted against percent CFTR function as measured by nasal potential difference (NPD). **(B)** Sweat chloride and evaporimetry data represented as percent normal sweat test from this study for COPD smokers (∇) and COPD former smokers (Δ) are interpolated and plotted on each curve. CF-PI = pancreatic insufficient CF; CF-PS = pancreatic sufficient CF [12,14].

To further clarify the relationship between CFTR testing methods, we compared sweat chloride and sweat secretion rate to CFTR function measured by NPD testing based on previously reported values among various CF phenotypes (Figure 5) [14]. While each curve is non-linear, each test is particularly sensitive at different extremes of phenotypic severity. Interpolating CFTR function (as measured by NPD) for the subjects enrolled in our study, we can estimate CFTR function in the various phenotypic groups, which varied depending on the assay used. Based on sweat chloride, the estimated CFTR function in COPD smokers was ~60% of normal whereas sweat evaporimetry estimated CFTR dysfunction was less severe (~90% of normal). Whether differences in the manner that cigarette smoking and COPD affect the secretory (sweat rate) versus absorptive (sweat chloride) compartments of the sweat gland has physiologic implications will require further study. Moreover, use of NPD represents only one of several methods to extrapolate relative CFTR function, thus estimates of absolute CFTR activity should be judged with caution.

The limitations of this observational cohort study include the relatively small numbers of patients in each cohort and the lack of age and gender matching despite similar age limits in the inclusion criteria. This concern is mitigated since the effect of COPD persists with use of multiple regression to account for these differences and consistency between two separate cohorts. Similarly, while age was related to sweat chloride abnormality on univariate analysis, it was not significant in the multiple regression model, suggesting the effect of smoking on sweat chloride was the dominant factor; moreover, sweat chloride was not related to age in a cohort of normal non-smokers. Given the heterogeneous nature of COPD, more study is needed to better classify disease phenotypes to allow directed therapeutics, and to further explore the etiology for the persistent decrement in CFTR function beyond the airway. Finally, our study provides further impetus to test whether therapeutics that directly or indirectly augment CFTR activity have a beneficial effect on patients with COPD, including those that have stopped smoking but exhibit a sustained CFTR defect. If successful, the results could have important implications in COPD therapeutics and open a new approach to address mucus stasis in the disease.

## Competing interests

SMR served as site PI for CF Clinical Trials sponsored by Vertex Pharmaceuticals. He has received COPD-related grant funding from NHLBI and Forest Research Institute. MTD has served on COPD-related advisory boards for Forest Pharmaceuticals, GlaxoSmithKline and Boehringer Ingelheim. He has served as site PI for contracted COPD clinical trials sponsored by GlaxoSmithKline and Boehringer Ingelheim. He has received COPD-related grant funding from NHLBI.

## Authors’ contributions

MTD and SMR conceived of the experiments; CC and ST conducted research; CC, BL, FJA, MTD and SMR analyzed the data; CC, MTD and SMR wrote the manuscript; MTD and SMR supervised the project. All authors read and approved the final manuscript.

## Supplementary Material

Additional file 1 Inclusion and Exclusion Criteria. Click here for file
