# Supplementary material for: Acquired defects in CFTR-dependent β-adrenergic sweat secretion in chronic obstructive pulmonary disease
Source: Respir Res. 2014 Feb 25;15(1):25. doi: 10.1186/1465-9921-15-25 (PMC4015030; doi:10.1186/1465-9921-15-25)
Supplement: Additional file 1 — Inclusion and Exclusion Criteria. [file 1465-9921-15-25-S1.docx]

Inclusion and Exclusion Criteria

Subjects age 40 – 75 years were eligible to participate. Spirometry requirements were a post-bronchodilator FEV_1_/FVC < 70% and FEV_1_ between 40 and 70% predicted for Groups 3 and 4 or normal lung function (FEV_1_/FVC > 70%, FVC > 80% and post-bronchodilator FEV_1_ > 80% predicted) for Groups 1 and 2. Subjects were excluded if they had another concomitant respiratory disorder; lung surgery with removal of a lobe or more, lung volume reduction, or lung transplantation; known or suspected active malignancy other than non-melanoma skin cancer; other illness expected to cause death within 12 months; pregnancy or suspected pregnancy; use of antibiotics and/or systemic steroids within the last month; unstable co-morbid illness including coronary artery disease (recent myocardial infarction or angina); decompensated heart failure; history of ventricular arrhythmias and/or pacemaker/defibrillator placement; change in concomitant medications in the month prior to enrollment; or adverse reaction to subcutaneous injection of beta adrenergic or cholinergic agonists.
